# Evaluation of Internal Medicine Physician or Multidisciplinary Team Comanagement of Surgical Patients and Clinical Outcomes: A Systematic Review and Meta-analysis

**DOI:** 10.1001/jamanetworkopen.2020.4088

**Published:** 2020-05-05

**Authors:** Margaret Shaw, Anita M. Pelecanos, Alison M. Mudge

**Affiliations:** 1 Royal Brisbane and Women’s Hospital, Herston, Queensland, Australia; 2 QIMR Berghofer Medical Research Institute, Brisbane, Queensland, Australia; 3 University of Queensland School of Clinical Medicine, Brisbane, Queensland, Australia

## Abstract

**Importance:**

Older patients who undergo surgery may benefit from geriatrician comanagement. It is unclear whether other internal medicine (IM) physician involvement improves outcomes for adults who undergo surgery.

**Objective:**

To evaluate the association of IM physician involvement with clinical and health system outcomes compared with usual surgical care among adults who undergo surgery.

**Data Sources:**

MEDLINE, Embase, CINAHL, and CENTRAL databases were searched for studies published in English from database inception to April 2, 2019.

**Study Selection:**

Prospective randomized or nonrandomized clinical studies comparing IM physician consultation or comanagement with usual surgical care were selected by consensus of 2 reviewers.

**Data Extraction and Synthesis:**

Data were extracted using the Preferred Reporting Items for Systematic Reviews and Meta-Analyses (PRISMA) guideline by 2 authors independently. Intervention characteristics were described using existing indicators. Risk of bias was assessed using Risk of Bias 2.0 and Risk of Bias in Nonrandomized Studies of Interventions tools. Studies were pooled when appropriate in meta-analysis using random-effects models. Prespecified subgroups included IM physician–only vs multidisciplinary team interventions and patients undergoing elective vs emergency procedures.

**Main Outcomes and Measures:**

The prespecified primary outcome was length of stay; other outcomes included 30-day readmissions, inpatient mortality, medical complications, functional outcomes, and costs.

**Results:**

Of 6027 records screened, 14 studies (with 1 randomized clinical trial) involving 35 800 patients (13 142 [36.7%] in intervention groups) were eligible for inclusion. Interventions varied substantially among studies and settings; most interventions described comanagement by a hospitalist or internist; 7 (50%) included a multidisciplinary team, and 9 (64%) studied predominantly patients who had elective procedures. Risk of bias in 10 studies (71%) was serious. Meta-analysis showed no significant association with length of stay (mean difference, −1.02 days; 95% CI, −2.09 to 0.04 days; *P* = .06) or mortality (odds ratio, 0.79; 95% CI, 0.56 to 1.11; *P* = .18), but multidisciplinary team involvement was associated with significant reduction in length of stay (mean difference, −2.03 days; 95% CI, −4.05 to −0.01 days; *P* = .05) and mortality (odds ratio, 0.67; 95% CI, 0.51 to 0.88; *P* = .004). There was no difference in 30-day readmissions (odds ratio, 0.89; 95% CI, 0.68 to 1.16; *P* = .39). Data could not be pooled for complications or costs. Only 1 study (7%) reported functional outcomes.

**Conclusions and Relevance:**

The findings of this study suggest that IM physician comanagement that includes multidisciplinary team involvement may be associated with reduced length of stay and mortality in adults undergoing surgery. Evidence was low quality, and well-designed prospective studies are still needed.

## Key Points

**Question:**

In adults who undergo inpatient surgery, does internal medicine (IM) physician involvement with or without a multidisciplinary team improve clinical or health service outcomes compared with standard surgical care?

**Findings:**

In this systematic review and meta-analysis of 14 studies (including 1 randomized clinical trial and a total of 35 800 patients), IM physician involvement alone was not associated with improved outcomes, but the involvement of an IM physician with a multidisciplinary team was associated with reduced length of stay and reduced inpatient mortality. Information about complications, functional decline, and costs was limited.

**Meaning:**

The findings of this study suggest that IM physician comanagement involving multidisciplinary teams may reduce length of stay and inpatient mortality among adults undergoing surgical procedures, but higher quality studies are needed.

## Teaser

This systematic review and meta-analysis evaluates the association of internal medicine physician involvement with clinical and health system outcomes compared with usual surgical care among adults who undergo surgery.

## Introduction

As surgical techniques advance, more medically complex patients have become candidates for surgical interventions, including those who are older, are frail, or have multiple comorbidities and are at higher risk for poor outcomes.^1,2^ The increase in the medical complexity of patients undergoing surgery has prompted increased involvement of internal medicine (IM) physicians in all aspects of perioperative care, including preoperatively optimizing the management of comorbidities, such as anemia, postoperatively managing complications, and maximizing functional recovery.^3^ Involvement of IM physicians (including internists and hospitalists) in surgical care is becoming increasingly proactive and coordinated rather than reactive, with IM physician involvement planned in either a consultative (eg, providing advice) or comanagement (ie, sharing decision-making and daily management) role as part of routine perioperative care.^4^ However, such models may be accompanied by additional costs and complexities, including additional investigations and the involvement of other multidisciplinary teams (MDTs), which include other nonmedical disciplines, such as nursing, physical therapy, social work, occupational therapy.^5^

Orthopedic surgery was the first surgical specialty to embrace proactive physician involvement in routine care.^6^ Orthogeriatrics is a rapidly expanding specialty, integrating geriatricians into the orthopedic team managing fractures in older patients. A 2010 literature review^7^ identified 21 studies encompassing 4 different models of orthogeriatric service, while a 2015 meta-analysis of randomized clinical trials (RCTs) for patients with hip fracture^8^ included 15 studies describing a range of models of integrated geriatric care. The meta-analysis concluded that a comprehensive geriatric care model was associated with greater functional improvement and an increased proportion of patients discharged back to their premorbid place of residence but found no significant difference in mortality or length of stay (LOS).^8^ Another systematic review^9^ examined geriatrician comanagement across different specialties, including elective orthopedic surgery and older general medical inpatients as well as patients with hip fracture. This study identified 6 RCTs and 6 quasi-experimental studies and found evidence for reduced LOS and reduced complications with geriatric comanagement. An international Delphi study^10^ recently developed quality structure and process indicators for inpatient geriatric comanagement programs, which are increasingly becoming the standard of care.

In contrast, the evidence supporting the value of IM physician involvement (including internists and hospitalists) in perioperative care of adults undergoing surgery, including younger adults, is limited. A 2017 systematic review focusing solely on preoperative consultation by IM physicians^11^ reported only 4 comparative studies (with 1 RCT), with inconsistent interventions and findings. Several retrospective observational studies have suggested that patients undergoing surgery who receive routine physician or hospitalist care have better outcomes, such as reduced LOS and mortality.^12,13,14,15^ However, others have indicated no difference or inconsistent associations with outcomes.^16,17^ Retrospective data has also suggested that the benefits of hospitalist intervention may be outweighed by increased costs.^18,19^ Thus, there is genuine uncertainty whether coordinated IM physician involvement in care of adults undergoing surgery is beneficial for patients and hospitals.

We planned and undertook a systematic review of published studies examining the role of IM physician involvement in care of adults undergoing surgery across a range of surgical specialties and the association of this care with clinical and health service outcomes, including LOS, complications, mortality, readmissions, functional outcomes, and costs of care.

## Methods

A protocol for this study was registered with PROSPERO. Reporting followed the Preferred Reporting Items for Systematic Reviews and Meta-analyses (PRISMA) reporting guideline.

### Eligibility Criteria

The review included studies with a prospective intervention group compared with a comparator group receiving usual surgical care, published in English in the peer-reviewed literature. The study population was adults (ie, aged >18 years) admitted as inpatients for major surgery (either emergent or elective) and returning postoperatively to the ward for management. Studies of patients admitted to the intensive care unit or discharged home immediately after the surgical procedure were excluded.

Interventions included preplanned postoperative involvement of an IM physician with or without an MDT and could include consultation, comanagement or shared care, or medical care with surgical consultation. The IM physician could be a general medical specialist, perioperative medicine specialist, internist, or hospitalist. Interventions led only by a geriatrician or anesthetist were excluded. Interventions consisting only of outpatient preoperative consultation were also excluded. The comparator included primary management by the surgical team without preplanned IM physician involvement but could include reactive medical consultation. Studies were required to report the primary outcome of LOS.

### Search Strategy and Information Sources

Search strings were developed with the aid of a librarian for MEDLINE, Embase, CINAHL, and CENTRAL databases and initially searched from database inception until August 21, 2017 (eAppendix in the Supplement), with an updated search using the same terms performed through April 2, 2019. Additional studies were identified from citation searching and relevant reviews. Scopus was used to search for studies that cited relevant papers identified in earlier searches.

### Study Selection

Database searching and title screening were conducted by 1 of us (M.S.). Abstract screening for inclusion was conducted by 1 of us (M.S.) and verified by a second (A.M.M.). Full-text screening for inclusion was conducted by 2 researchers (M.S. and A.M.M.) independently. Discrepancies on whether a study met inclusion criteria were resolved by consensus.

### Data Extraction and Synthesis

Data were extracted from published studies using planned data fields by 2 investigators (M.S. and A.M.M.) independently. Data were collected on study design, time frame, population inclusions and exclusions (eg, specific screening criteria, such as age), and details of care in both the intervention and comparator groups, including type of physician leading the team (eg, internist, hospitalist), team communication (eg, frequency of meetings), role of IM physician and MDT (eg, comanagement or consultation, use of protocols and order sets, discharge planning), and all reported outcomes, using the original paper’s descriptions. A total of 8 study authors were contacted by email to seek missing data, including measures of variance or unreported outcomes; 1 author supplied additional data.

Risk of bias was independently assessed by 2 investigators (M.S. and A.M.M.). For RCTs the Risk of Bias 2.0 tool^20^ was used. For nonrandomized studies, bias risk was assessed using the Risk of Bias in Nonrandomized Studies of Interventions tool.^21^ Discrepancies in assessment were resolved by consensus.

### Statistical Analysis

Study characteristics were tabulated and features of the interventions were summarized using quality indicators adapted from those published for geriatric comanagement models.^10^ Outcome data were tabulated for each study when available. When outcomes were available for a sufficient number of studies, data were entered into RevMan version 5.3 (Cochrane Training) for meta-analysis. Outcomes available for 3 or fewer studies are discussed in text. Randomized and nonrandomized studies were not pooled to minimize methodological heterogeneity. Preplanned subgroups included predominantly (ie, >50%) patients undergoing emergent procedures vs predominantly patients undergoing elective procedures, and interventions including IM physician–led MDT vs IM physician–only interventions. In nonrandomized studies, statistically adjusted outcome estimates were analyzed separately from unadjusted outcome estimates. Where standard deviation for LOS was not available, it was calculated from confidence intervals or from *P* values when confidence intervals were not reported.^22^ In 1 study^23^ that analyzed LOS as a Poisson distributed outcome, the standard deviation was calculated using the square root of the mean, per the properties of the Poisson distribution. Random-effects models were used for all analyses with inverse-variance weighting methods, except when event rates were very low (eg, mortality data), in which case we used the Mantel-Haenszel method. We calculated mean differences with 95% CIs for LOS, and odds ratios (ORs) with 95% CIs for mortality and 30-day readmissions. Data were displayed in forest plots, and heterogeneity was assessed using *I*^2^ statistics, with an *I*^2^ greater than 50% indicating substantial heterogeneity. We inspected for publication bias using funnel plots. We considered a 2-tailed *P* < .05 statistically significant.

## Results

**Figure 1.**
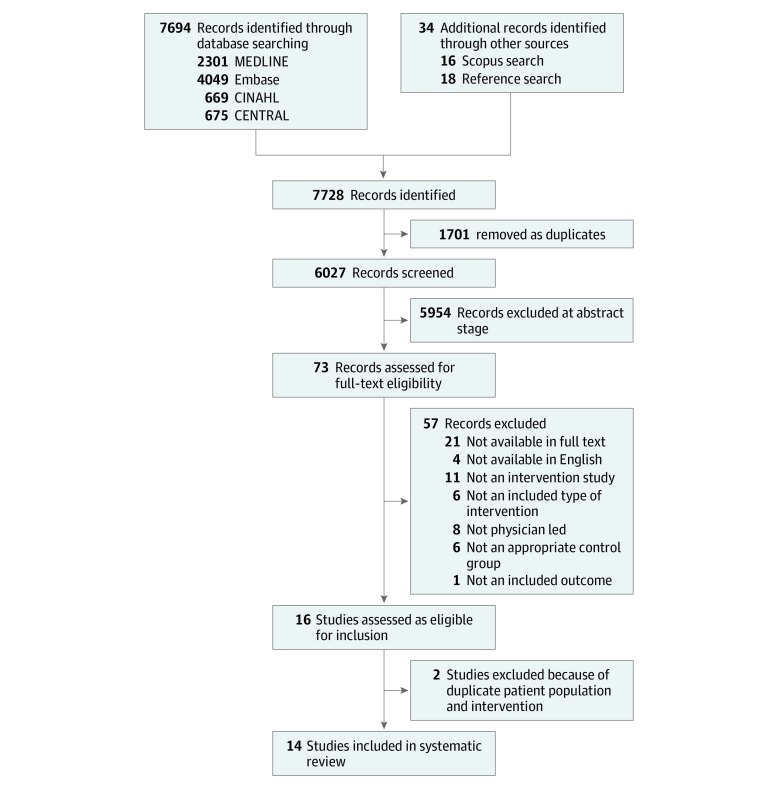
Study Selection Flowchart

Figure 1 outlines the process of study screening and selection for inclusion in this review. Of 6027 articles identified in initial searches, 73 underwent full-text assessment and 16 studies were identified for inclusion, including 1 (6%) RCT,^24^ 1 (6%) comparative cohort study,^25^ and 14 (88%) pre-post studies,^23,26,27,28,29,30,31,32,33,34,35,36,37,38^ 3 (21%) of which included a concurrent control group.^26,32,33^ A total of 3 pre-post studies (21%) were conducted at the same site using the same intervention but with different study dates and inclusion criteria,^27,34,35^ so we included the study with the longest duration and largest range of outcomes.^27^ Study characteristics for the final 14 studies (including 35 800 patients; 13 142 participants [36.7%] in intervention groups and 22 658 participants [63.3%] in control groups) are summarized in Table 1 and details of the structure and process of the interventions in eTable 1 in the Supplement. Overall, 11 studies (79%) were from the US,^23,24,25,26,27,28,31,32,36,37,38^ 2 (14%) from the same investigator group in Spain,^29,30^ and 1 (7%) from Canada^33^; 6 (43%) studies were in orthopedic patients, and other specialties included neurosurgery, vascular surgery, colorectal surgery, thoracic surgery, ophthalmology, otolaryngology, and trauma surgery. A total of 5 studies (36%) were confined to emergency admissions only (hip fracture or trauma),^23,25,33,36,38^ 2 (14%) included elective admissions only,^24,31^ and others included a mix of both emergency and elective cases, with 9 (64%) studying predominantly elective inpatients. Overall, 12 studies (86%) were considered comanagement, 1 (7%) involved a comprehensive multidisciplinary program, of which 1 facet was the involvement of a consulting internist and/or geriatrician,^36^ and 1 (7%) involved a primary medical service caring for patients undergoing trauma surgery and requesting surgical consultation if required.^23^ A total of 5 studies (36%) involved an internist,^28,29,30,36,38^ and the remainder involved hospitalists; 7 studies (50%) reported the involvement of an MDT.^25,27,32,36,37,38^ Overall, 6 studies (43%) had specific inclusion criteria for the service,^23,24,26,27,31,36^ and 5 studies (36%) explicitly included preoperative assessment.^24,28,31,33,38^

**Table 1.** Characteristics of Studies Included in Review

Source (country)	Study design and setting	Surgery type and intervention	Intervention population			Comparator population		
			No. (% men)	Age, mean (SD), y	Elective, %	No. (% men)	Age, mean (SD), y	Elective, %
Zuckerman et al,^36^ 1992 (US)	Pre-post in an urban tertiary care hospital	Orthopedic internist with MDT	431 (19.0)	80.4 (NR)	0	60 (21.7)	80.3 (NR)	0
Macpherson et al,^28^ 1994 (US)	Pre-post in a university-affiliated VA hospital	Thoracic internist	78 (98.7)	63.1 (NR)	NR	86 (100)	63.1 (NR)	NR
Huddleston et al,^24^ 2004 (US)	Randomized clinical trial in an academic medical center	Orthopedic hospitalist	232 (45.3)	72.6 (10.6)	100	237 (47.3)	73.7 (8.7)	100
Pinzur et al,^31^ 2009 (US)	Pre-post in an academic medical center	Orthopedic hospitalist	86 (48.8)	51.5 (NR)	100	54 (33.3)	54.3 (NR)	100
Salottolo et al,^23^ 2009 (US)	Pre-post in a community trauma center	Trauma hospitalist	261 (NR)	72.0 (14.9)	0	239 (NR)	67.4 (18.3)	0
Auerbach et al,^26^ 2010 (US)	Pre-post, control in an academic medical center	Neurosurgery hospitalist	3393 (49.2)	54.0 (15.6)	67.5	4203 (47.5)	53.1 (15.6)	66.5
Della Rocca et al,^38^ 2013 (US)	Pre-post in an academic medical center	Orthopedic internist with MDT	115 (22.6)	82 (NR)	0	31 (29.0)	82 (NR)	0
Montero-Ruiz et al,^30^ 2015a (Spain)	Pre-post in an academic medical center	Otolaryngology internist	642 (57.6)	49.9 (NR)	81.19	987 (58.6)	45.3 (NR)	86.2
Montero-Ruiz et al,^29^ 2015b (Spain)	Pre-post in an academic medical center	Ophthalmology internist	244 (60.7)	64.6 (NR)	86.1	345 (54.8)	63.6 (NR)	81.4
Iberti et al,^27^ 2016 (US)	Pre-post in an urban tertiary care hospital	Vascular hospitalist with MDT	1487 (58.6)	64.6 (NR)	62.1	944 (57.5)	65.9 (NR)	63.5
Noticewala et al,^25^ 2016 (US)	Comparative cohort in an academic trauma center and community hospital	Orthopedic hospitalist with MDT	129 (20.2)	84.5 (11.5)	0	138 (32.6)	79.9 (10.8)	0
Rohatgi et al,^32^ 2016 (US)	Pre-post, control in an academic medical center	Orthopedic and neurosurgery hospitalist with MDT	4650 (NR)	58.5 (16.8)^a^	71.3^a^	14 156 (NR)	57.8 (17.2)^a^	71.7^a^
Soong et al,^33^ 2016 (Canada)	Pre-post, control in an academic medical center	Orthopedic hospitalist with MDT	331 (28.7)	79.4 (13.7)	0	240 (30.8)	80.1 (13.0)	0
Rohatgi et al,^37^ 2018 (US)	Pre-post in an academic medical center	Colorectal hospitalist with MDT	1062 (54.5)	54.8 (16.5)	77.8	938 (50.0)	54.1 (17.8)	77.6

Abbreviations: MDT, multidisciplinary team; NR, not reported; VA, Veterans Affairs.

^a^ Additional data obtained from author.

### Risk of Bias Assessment

The assessment of bias risk for included studies for the objectively assessed outcomes of LOS, in-hospital mortality, and readmissions are summarized in eTable 2 in the Supplement. The only study with a low risk of bias was the RCT^24^; all quasi-experimental studies had at least a moderate risk of bias, most commonly because of confounding, selection bias, and deviation from intended intervention. We did not identify prepublished protocols for any study, making reporting bias difficult to judge. Of the 13 quasi-experimental studies, 3 (23%) were considered to have a moderate risk of bias. Auerbach et al^26^ used a concurrent control group to reduce risk of confounding, and Rohatgi et al used a concurrent control group with propensity scoring and difference-in-difference design in a study among orthopedic and neurosurgery patients^32^ and multivariable regression models in a study of colorectal patients.^37^ Salottolo et al^23^ also controlled for confounding using a multivariable model; however, the historical control group used was constructed retrospectively using an algorithm, which gave an increased risk of selection bias and serious risk of bias because of deviation from intended interventions. All other studies (10 [71%]) were considered at serious risk of bias.

### LOS

**Figure 2.**
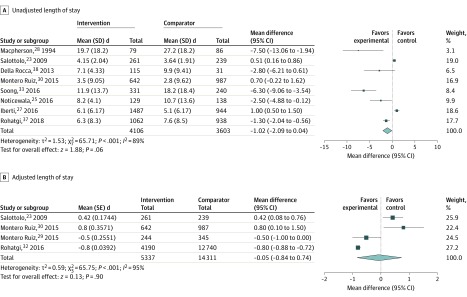
Forest Plot for Unadjusted and Adjusted Length of Stay

Patient LOS was reported by all studies, although a range of different reporting methods were used, and measures of variance were often absent (Table 2). In the only RCT, Huddleston et al^24^ reported no significant difference in LOS, although when they included discharge delay in their definition of LOS, a shorter mean LOS was reported (mean difference, −0.5 days; 95% CI, −0.8 to −0.1 days). In the nonrandomized studies, 5 (38%) reported a significant association with reduction in unadjusted mean LOS,^25,28,33,37,38^ 2 (15%) reported a significant increase,^23,27^ and 4 (31%) reported no change^26,29,30,36^; 2 (15%) did not report unadjusted mean LOS.^31,32^ Overall 8 nonrandomized studies (62%) representing 7709 patients had data that could be included in an unadjusted LOS meta-analysis, and 4 studies (31%) representing 19 648 patients could be included in an adjusted LOS meta-analysis, as shown in Figure 2. The intervention was not associated with unadjusted LOS (mean difference, −1.02 days; 95% CI, −2.09 to 0.04 days; *P* = .06) or when studies including adjusted LOS were used (adjusted mean difference, −0.05 days; 95% CI, −0.84 to 0.74 days; *P* = .90). There was very high heterogeneity in the unadjusted meta-analysis (*I*^2^ = 89%). In subgroup analyses of unadjusted LOS (eFigure 1 and eFigure 2 in the Supplement), interventions that included an MDT were associated with reduced mean LOS (mean difference, −2.03 days; 95% CI, −4.05 to −0.01; *P* = .05), but physician-only models were not associated with reduced mean LOS (mean difference, 0.21 days; 95% CI, −1.05 to 1.48 days; *P* = .74). No association was seen in elective or emergency subgroups. Heterogeneity remained high in all subgroups. Examination of the funnel plot for all studies included in the unadjusted LOS meta-analysis indicated that studies with larger standard errors seemed to have a larger reduction in LOS, although this pattern was unclear because of the small number of studies included (eFigure 3 in the Supplement).

**Table 2.** Summary of Major Outcomes

Source	Mean length of stay among intervention vs comparator, d		In-hospital mortality among intervention vs comparator, No./total No. (%)	30 d readmissions among intervention vs comparator, No./total No. (%)	Any medical complication among intervention vs comparator, No./total No. (%)	Costs among intervention vs comparator, US $
	Unadjusted	Adjusted				
Zuckerman et al,^36^ 1992	23.2 vs 27.7	NA	25/431 (5.8) vs 3/60 (5.0)	NA	162/431 (37.6) vs 39/60 (65.0)	NA
Macpherson et al,^28^ 1994	19.7 vs 27.2; mean difference, −7.5; 95% CI, −1.9 to −13.1	NA	2/79 (2.5) vs 7/86 (8.1)	3/79 (3.8) vs 3/86 (3.5)	NA	NA
Huddleston et al,^24^ 2004	5.6 vs 5.7; mean difference, −0.1; 95% CI, −0.5 to 0.2	NA	0/232 vs 1/237 (0.4)	NA	89/232 (38.4) vs 119/237 (50.2)	Mean direct medical costs per patient, 15 373 vs 15 283
Pinzur et al,^31^ 2009	NA	O/E ratio, 0.693 vs 0.862	NA	NA	NA	O/E cost of hospital care, 0.684 vs 0.699
Salottolo et al,^23^ 2009	4.2 vs 3.6	4.1 vs 3.7	2/261 (0.8) vs 4/239 (1.7); adjusted OR, 0.41; 95% CI, 0.07 to 2.26	NA	9.8% vs 10.5%; adjusted OR, 0.86; 95% CI, 0.73 to 1.02	NA
Auerbach et al,^26^ 2010	5.0 (IQR, 3.8) vs 5.0 (IQR, 3.8)^a^	Adjusted rate ratio, 0.97; 95% CI, 0.92 to 1.03	88/3393 (2.6) vs 104/4203 (2.5); adjusted OR, 0.97; 95% CI, 0.65 to 1.05	192/3393 (5.7) vs 277/4203 (6.6); adjusted OR, 0.83; 95% CI, 0.65 to 1.05	NA	24 533 (IQR, 15 881 to 41 943) vs 23 867 (IQR, 15 133 to 40 966); adjusted rate ratio 0.94 (95% CI, 0.88 to 1.00)^a^
Della Rocca et al,^38^ 2013	7.1 (95% CI, 6.3 to 7.9) vs 9.9 (95% CI, 6.4 to 13.3)	NA	5/115 (4.3) vs 3/31 (9.7)	16/115 (13.9) vs 6/31 (19.4)	NA	Mean costs per patient, 38 586 (95% CI, 35 210 to 41 963) vs 52 323 (95% CI, 31 641 to 77 006)
Montero Ruiz et al,^30^ 2015a	3.5 (95% CI, 2.8 to 4.2) vs 2.8 (95% CI, 2.2 to 3.4); mean difference, 0.7; 95% CI, −0.2 to 1.7	Mean adjusted difference, −0.8; 95% CI, −1.6 to −0.1	1.2% (95% CI, 0.4% to 2.1%) vs 0.3% (95% CI, 0% to 0.6%)	3.0% (95% CI, 1.6% to 4.3%) vs 2.8% (95% CI, 1.8% to 3.9%)^c^	NA	Estimated mean cost savings per hospitalization, €258.40
Montero Ruiz et al,^29^ 2015b	1.5 (95% CI, 1.1 to 1.8) vs 1.8 (95% CI, 1.4 to 2.1); median difference, –0.3; 95% CI, −0.8 to 0.2^a^	Mean adjusted difference, −0.5; 95% CI, –1.0 to 0.01	0% vs 0%	4.5% (95% CI, 1.9% to 7.1%) vs 3.2% (95% CI, 1.3% to 5.0%)^b^	NA	Estimated mean cost savings per hospitalization, €161.50
Iberti et al,^27^ 2016	6.1 vs 5.1	O/E ratio, 0.88 vs 0.83	1.00% vs 2.01%	20.6% vs 21.9%	NA	NA
Noticewala et al,^25^ 2016	8.2 (SD, 4.1) vs 10.7 (SD, 13.6)	NA	4/129 (3.1) vs 2/138 (1.4)	NA	NA	NA
Rohatgi et al,^32^ 2016	NA	Propensity score adjusted, 4.6 (SD, 2.2) vs 5.4 (SD, 2.2)	40/4650 (0.9) vs 173/14156 (1.2)^c^	1.9% vs 3.4%; propensity score adjusted, 1.8% vs 3.0%; OR, 0.63; 95% CI, 0.54 to 0.90	8.2% vs 10.1%; propensity score adjusted; 8.0% vs 9.5%; OR, 0.83; 95% CI, 0.70 to 0.95^d^	Estimated mean cost savings per patient, 2642 to 4303
Soong et al,^33^ 2016	11.9 (SD, 13.7) vs 18.2 (SD, 18.4)	NA	7/331 (2.1) vs 12/240 (5.0)	20/331 (6.0) vs 11/240 (4.6)	NA	Total mean (SD) hospital costs per hospitalization, CAD $13 755 (CAD $11 899) vs CAD $18 706 (CAD $25 198)
Rohatgi et al,^37^ 2018	6.3 (SD, 8.3) vs 7.6 (SD, 8.5)^c^	Adjusted RR for length of stay >5 d, 0.73; 95% CI, 0.64 to 0.83	5/1062 (0.5) vs 4/938 (0.4)^c^	172/1062 (16.1) vs 122/938 (13.0); adjusted RR, 1.87; 95% CI, 1.00 to 3.64	96/1062 (9.0) vs 95/938 (10.1); adjusted RR, 0.96; 95% CI, 0.74 to 1.24	Median direct cost of care, −10.3%

Abbreviation: IQR, interquartile range; NA, not applicable; O/E, observed/expected; OR, odds ratio; RR, relative risk.

^a^ Median reported.

^b^ 15-day readmission rate reported.

^c^ Additional data obtained from author.

^d^ Outcome defined as patients with more than 1 complication.

### Mortality

**Figure 3.**
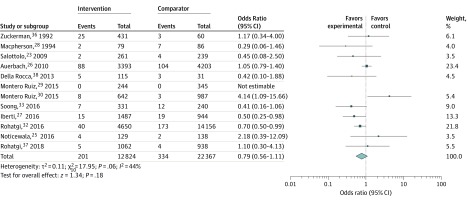
Forest Plot for In-Hospital Mortality

In-hospital mortality was available for 13 studies (Table 2). Huddleston et al^24^ reported no difference between groups, but there was only 1 death reported. Among 12 nonrandomized studies, 7 studies (58%) reported reduced mortality in the intervention group (1 [8%] with a statistically significant difference^27^) and 5 studies (42%) reported higher mortality in the intervention group (1 [8%] with a statistically significant difference^30^). A meta-analysis of the nonrandomized studies, which included 35 191 patients, is shown in Figure 3. The intervention was not associated with reduced mortality (OR, 0.79; 95% CI, 0.56-1.11; *P* = .18). One study (8%) had 0 mortality in both groups, raising methodologic issues in dealing with 0 events in a meta-analysis. To further support our mortality results, no difference in findings resulted from repeating the analysis using the inverse-variance method with a 0.5 continuity correction for 0 events. A funnel plot revealed no discernible patterns (eFigure 3 in the Supplement). There was moderate statistical heterogeneity (*I*^2^ = 44%). In subgroup analysis, MDT interventions were associated with reduced mortality (OR, 0.67; 95% CI, 0.51-0.88; *P* = .004) with no heterogeneity (*I*^2^ = 0%), but no association was seen in the physician-only subgroup (OR, 0.98; 95% CI, 0.40-2.41; *P* = .96) (eFigure 4 in the Supplement). There was no significant association with mortality in the elective or emergency subgroups (eFigure 5 in the Supplement).

### Readmissions

Readmissions at 30 days were not reported in the RCT, but data were available for 7 nonrandomized studies (54%), including 31 715 patients.^26,27,28,32,33,37,38^ Rohatgi et al^32^ reported a significant association with reduced readmissions, with no association reported in the other studies (Table 2). Two additional studies reported 15-day readmissions, with no association between the intervention and this outcome. In meta-analysis, there was no association of the intervention with 30-day readmissions (OR, 0.89; 95% CI, 0.68-1.16; *P* = .39) (eFigure 6 in the Supplement). There was substantial heterogeneity (*I*^2^ = 78%), with insufficient studies to undertake meaningful subgroup analyses. Funnel plot analysis showed no discernable patterns (eFigure 3 in the Supplement).

### Complications

Data for medical complications were reported in 5 studies (36%),^23,24,32,36,37^ with 3 (60%) reporting a statistically significant decrease in complications associated with the intervention^24,32,36^ (Table 2). The small number of studies and variation in definition of complications precluded a meaningful meta-analysis of this outcome.

### Cost

Cost data were reported by 9 studies (64%), of which 4 (44%) reported direct measures of hospital and physician charges (Table 2). There was no difference in the 1 RCT (11%),^24^ and 3 studies (33%) had a potential association between the intervention and cost savings.^26,33,37^ Three studies (33%) imputed costs from LOS^29,30,32^ and did not present the additional resource requirements for the comanagement model. One study (11%) reported only the observed-to-expected cost ratio for intervention and control groups.^31^ Variation in methods and reporting precluded meaningful synthesis of cost data.

### Other Outcomes

Only 1 study (7%) among patients with hip fractures^36^ reported functional outcomes, demonstrating a significant association of the intervention with greater independence and lower nursing home placement. Two other studies (14%) among patients with hip fractures reported no associations with admissions to rehabilitation or skilled nursing facilities.^23,33^ Three studies (21%) reported lower intensive care use postoperatively^36,38^ or following rapid response activations.^37^ Four studies (29%) reported measures of patient satisfaction with the intervention,^24,26,32,37^ with 1 study (7%) showing a statistically significant association with some measures of satisfaction but not overall hospital experience or likelihood of recommending the hospital.^26^ The other studies showed no significant findings in satisfaction. Four studies (29%) reported on provider satisfaction with the intervention,^24,26,27,32^ with 1 study (7%) reporting a significant association of the intervention with greater nursing satisfaction^26^; the other studies reported favorable provider impression of the intervention without statistical testing.

## Discussion

To our knowledge, this is the first systematic review and meta-analysis of physician involvement in surgical care in the adult population to extend beyond geriatrician-led interventions. Our review demonstrated that the quality of evidence remains low, despite many institutions adopting IM physician comanagement of surgical procedures.^6^ We identified 1 RCT, conducted among relatively low-risk elective joint replacement patients. We also identified 13 quasi-experimental studies, of which 3 had moderate risk of bias and the remainder had serious risk of bias. Common reasons for bias included selection bias, confounding, potential selective reporting, and potential deviation from intended intervention.

Interventions varied substantially among studies. Although most studies specified a comanagement model with daily IM physician availability, there was variation in patient selection, physician type, involvement of other MDT members, and the responsibilities of the medical and surgical staff.

The single RCT was conducted among elective orthopedic patients and showed no significant difference in LOS or mortality; however, it did report reduced medical complications. After synthesizing data from nonrandomized studies, there was low-quality evidence that interventions were not significantly associated with changes in LOS, mortality, or readmissions. However, IM physician–led multidisciplinary models were significantly associated with reductions in LOS and mortality. These findings support evidence from geriatric comanagement models (mainly among patients with hip fractures). Meta-analyses have shown varied findings regarding reduced mortality and LOS in those models, but studies that have specifically explored different intervention designs suggest that greater integration (ie, comanagement rather than consultation) and involvement of the MDT (ie, nonmedical professionals) are associated with significant improvements in mortality and/or LOS.^7,9,39^

Marked variation in the measurement of medical complications and costs made it difficult to draw meaningful conclusions about these measures. There were very limited data on functional outcomes, all confined to hip fracture studies.

Comanagement models, in which physicians contribute directly to the management of surgical cases, offer a number of potential advantages for patients and health systems. At the patient level, physicians may recognize, predict, and mitigate problems associated with medical comorbidities unrecognized by the surgical team, provide advice for preoperative optimization, and facilitate a holistic perspective of risks and benefits relevant to patient goals in shared surgical decision-making. At the ward level, they may also contribute to evidence-based guidelines and order sets for medical issues to ensure best-practice care, coordinate care and discharge planning with the MDT, coordinate other specialist consultations, and contribute to multidisciplinary quality improvement initiatives.^32,40,41^ This suggests a role well beyond clinical consultation and may explain why these complex interventions appear to show more promise than preoperative physician consultation as a single strategy.^11^ However, this complexity also leads to potential variability within and between services and reliance on good communication and teamwork.^41^ Evaluation designs, such as cluster randomization or well-conducted interrupted time series designs, would be appropriate for these complex interventions^42^ but were not represented in our review. Measurement should also include costs and benefits beyond direct medical costs and medical complications, such as changes in team functioning^43^ and focusing on outcomes of importance to patients.^44^

### Limitations

This study has limitations. The inclusion of diverse interventions and patient populations undergoing surgery necessitated the use of random-effects models for meta-analysis, and this approach can provide conservative estimates and wide confidence intervals, which may underestimate intervention effects. Search strings were carefully developed to capture as many potentially relevant studies as possible, but given the variable terminology used worldwide to describe IM physicians as well as variable descriptors of physician-led interventions, there is a possibility that we missed important studies in our search. We only included English-language publications. Findings from the review were limited by the risk of bias of most individual studies, although the largest nonrandomized studies also had the lowest risk of bias.^26,32^ Additional challenges to collating the data included inconsistency in outcome definitions and measurement; variable statistical reporting of point estimates and measures of variation in LOS, which limited the number of studies which could be included in meta-analysis; and variation in adjustment factors used in adjusted analyses. None of the included studies had a published protocol, so there were uncertain risks of selective reporting. The only RCT was a physician-only intervention in a low-risk elective group, which may have limited the potential effect size and generalizability of findings. The overall quality of the evidence is low to very low, with substantial heterogeneity only partly explained by the specified subgroups. Future analyses might explore the association of other variables (eg, surgical type, physician type, patient age, intervention components) with outcomes, but we have not explored these further, given the relatively small number of studies currently available. Most studies were conducted in the US, where health system organization and funding differ from other parts of the world. There was very limited reporting of important patient-centered outcomes, such as functional status and discharge destination.

## Conclusions

This systematic review and meta-analysis of IM physician involvement in surgical care in the adult population did not show significant associations with clinical or health service outcomes. Benefits varied among different models, with MDT models showing a significant association with reduced LOS and mortality. The overall quality of evidence was low, and well-designed prospective studies appropriate for system-level interventions (such as cluster randomized designs) with well-specified and generalizable interventions in high-risk patient groups would be valuable to inform practice and policy. Consistent approaches to measuring complications, functional outcomes, and costs would provide substantial additions to the existing literature.

## Notes

Supplement. **eAppendix.** Example Search String **eTable 1. **Intervention Characteristics Described in Studies in Review, Based on Indicators Developed for Geriatric Comanagement Models **eTable 2. **Risk of Bias for Included Studies for the Outcomes of Length of Stay, Mortality, and Readmissions **eFigure 1. **Forest Plot for Unadjusted Length of Stay by Physician-Only or Multidisciplinary Team (MDT) Model **eFigure 2. **Forest Plot for Unadjusted Length of Stay by Elective or Emergency Status **eFigure 3. **Funnel Plots for Length of Stay, Mortality, and Readmissions **eFigure 4. **Forest Plot for In-Hospital Mortality by Physician-Only or Multidisciplinary Team (MDT) Model **eFigure 5. **Forest Plot for In-Hospital Mortality by Elective or Emergency Status **eFigure 6. **Forest Plot for 30-Day Readmissions
