# Supplementary material for: Evaluation of Internal Medicine Physician or Multidisciplinary Team Comanagement of Surgical Patients and Clinical Outcomes: A Systematic Review and Meta-analysis
Source: JAMA Netw Open. 2020 May 5;3(5):e204088. doi: 10.1001/jamanetworkopen.2020.4088 (PMC7201311; doi:10.1001/jamanetworkopen.2020.4088)
Supplement: Supplement. — eAppendix. Example Search String eTable 1. Intervention Characteristics Described in Studies in Review, Based on Indicators Developed for Geriatric Comanagement Models eTable 2. Risk of Bias for Included Studies for the Outcomes of Length of Stay, Mortality, and Readmissions eFigure 1. Forest Plot for Unadjusted Length of Stay by Physician-Only or Multidisciplinary Team (MDT) Model eFigure 2. Forest Plot for Unadjusted Length of Stay by Elective or Emergency Status eFigure 3. Funnel Plots for Length of Stay, Mortality, and Readmissions eFigure 4. Forest Plot for In-Hospital Mortality by Physician-Only or Multidisciplinary Team (MDT) Model eFigure 5. Forest Plot for In-Hospital Mortality by Elective or Emergency Status eFigure 6. Forest Plot for 30-Day Readmissions [file jamanetwopen-3-e204088-s001.pdf]

Supplementary Online Content

Shaw M, Pelecanos A, Mudge AM. Evaluation of internal medicine physician or multidisciplinary team comanagement of surgical patients and clinical outcomes. *JAMA Netw Open*. 2020;3(5):e204088. doi:10.1001/jamanetworkopen.2020.4088

eAppendix. Example Search String
eTable 1. Intervention Characteristics Described in Studies in Review, Based on Indicators Developed for Geriatric Comanagement Models
eTable 2. Risk of Bias for Included Studies for the Outcomes of Length of Stay, Mortality, and Readmissions
eFigure 1. Forest Plot for Unadjusted Length of Stay by Physician-Only or Multidisciplinary Team (MDT) Model
eFigure 2. Forest Plot for Unadjusted Length of Stay by Elective or Emergency Status
eFigure 3. Funnel Plots for Length of Stay, Mortality, and Readmissions
eFigure 4. Forest Plot for In-Hospital Mortality by Physician-Only or Multidisciplinary Team (MDT) Model
eFigure 5. Forest Plot for In-Hospital Mortality by Elective or Emergency Status
eFigure 6. Forest Plot for 30-Day Readmissions

This supplementary material has been provided by the authors to give readers additional information about their work.

© 2020 Shaw M et al. *JAMA Network Open.*

eAppendix. Example Search String

Clinical Question: In adult surgical patients, does internal medicine (IM) physician involvement with or without a multidisciplinary team improve clinical or health service outcomes when compared to standard surgical care?

MEDLINE

	**Search string**	**Results**
1	TI surgery OR AB surgery OR TI surgical OR AB surgical OR TI surgeon OR AB surgeon OR MM "postoperative care" OR MM "postoperative complications" OR MM "postoperative period" OR MM "perioperative care" OR MM “preoperative care” OR MM “preoperative period”	**1,790,187**
2	TI hospitalist* OR AB hospitalist* OR TI internist* OR AB internist* OR TI physician OR AB physician OR TI "general medicine" OR AB "general medicine" OR TI “perioperative medicine” OR AB “perioperative medicine” OR MM hospitalists OR MM "internal medicine"	**373,912**
3	TI comanagement OR AB comanagement OR TI co-management OR AB co-management OR TI "shared care" OR AB "shared care" OR TI consult OR AB consult OR TI consultant OR AB consultant OR TI consultation OR AB consultation OR MM "Referral and Consultation" OR MM “Models, Organizational”	**116,213**
4	1 AND 2 AND 3	**2,301**

© 2020 Shaw M et al. *JAMA Network Open.*

eTable 1. Intervention Characteristics Described in Studies in Review, Based on Indicators Developed for Geriatric Comanagement Models[1]

			**Structure indicators**							**Process indicators**						
**Study**	**Specialty**	**Physician**	**MDT**	**Daily availability**	**Regular QI**	**Selection Screening**	**Inclusion criteria**	**Evidence-based protocols**	**Standard order sets**	**Co-manage-ment**	**Daily rounds**	**MDT meeting**	**Advance care planning**	**Discharge planning**	**Pre-op assess-ment**
Zuckerman (1992)[2]	Orthopaedic	Internist[a]	✓			✓	Age 65+, premorbid status					✓ weekly			
Macpherson (1994)[3]	Thoracic	Internist		✓			All patients			✓	✓			✓	✓
Huddleston (2004)[4]	Orthopaedic	Hospitalist		✓		✓	Age 75+, co-morbidities		✓	✓	✓			✓	✓
Pinzur (2009)[5]	Orthopaedic	Hospitalist		✓		✓	Co-morbidity or social issue	✓	✓	✓	✓			✓	✓
Salottolo (2009)[6]	Trauma	Hospitalist		✓	✓	✓	11 exclusion criteria	✓			✓				
Auerbach (2010)[7]	Neurosurgery	Hospitalist		✓		✓	Comorbidities, high risk for complications		✓	✓	✓				
Della Rocca (2013)[8]	Orthopaedic	Internist[b]	✓	✓	✓		Age 65+	✓	✓	✓	✓	✓ unclear		✓	✓
Montero Ruiz (2015a)[9]	Otolaryngology	Internist		✓			All patients			✓					
Montero Ruiz (2015b)[10]	Ophthal-mology	Internist		✓			All patients			✓					
Iberti (2016)[11]	Vascular	Hospitalist	✓	✓	✓	✓	ASA 3+, LOS >1 day			✓	✓	✓ daily			
Noticewala (2016)[12]	Orthopaedic	Hospitalist	✓	✓			All patients		✓	✓	✓	✓ daily			
Rohatgi (2016)[13]	Orthopaedic & Neurosurgery	Hospitalist	✓	✓	✓		All patients	✓	✓	✓	✓	✓ daily		✓	✓

© 2020 Shaw M et al. *JAMA Network Open.*

Soong (2016)[14]	Orthopaedic	Hospitalist	✓	✓	✓		All patients	✓	✓	✓	✓	✓ daily	✓ family meetings	✓	✓
Rohatgi (2018)[15]	Colorectal	Hospitalist	✓	✓	✓		All patients	✓	✓	✓	✓	✓ daily		✓	✓

MDT multidisciplinary team; QI quality improvement; ASA American society of anaesthesiologist physical status classification class; LOS length of stay
[a] model also included geriatricians
[b] model also included geriatricians and family practitioners

1. Van Grootven B, McNicoll L, Mendelson DA, et al. Quality indicators for in-hospital geriatric co-management programmes: a systematic literature review and international Delphi study. *BMJ Open.* 2018;8(3):e020617.
2. Zuckerman JD, Sakales SR, Fabian DR, Frankel VH. Hip fractures in geriatric patients: Results of an interdisciplinary hospital care program. *Clin Orthop Relat Res.* 1992(274):213-225.
3. Macpherson DS, Parenti C, Nee J, Petzel RA, Ward H. An internist joins the surgery service: does comanagement make a difference? *J Gen Intern Med.* 1994;9(8):440-444.
4. Huddleston JM, Long KH, Naessens JM, et al. Medical and surgical comanagement after elective hip and knee arthroplasty: a randomized, controlled trial. *Ann Intern Med.* 2004;141(1):28-38.
5. Pinzur MS, Gurza E, Kristopaitis T, et al. Hospitalist-orthopedic co-management of high-risk patients undergoing lower extremity reconstruction surgery. *Orthop.* 2009;32(7):495-495.
6. Salottolo K, Slone DS, Howell P, et al. Effects of a nonsurgical hospitalist service on trauma patient outcomes. *Surg.* 2009;145(4):355-361.
7. Auerbach AD, Wachter RM, Cheng HQ, et al. Comanagement of surgical patients between neurosurgeons and hospitalists. *Arch Intern Med.* 2010;170(22):2004-2010.
8. Della Rocca GJ, Moylan KC, Crist BD, Volgas DA, Stannard JP, Mehr DR. Comanagement of geriatric patients with hip fracture: a retrospective, controlled, cohort study. *Geriatr Orthop Surg Rehabil.* 2013;4(1):10-15.
9. Montero Ruiz E, Rebollar Merino Á, Rivera Rodríguez T, García Sánchez M, Agudo Alonso R, Barbero Allende JM. Effect of comanagement with internal medicine on hospital stay of patients admitted to the Service of Otolaryngology. *Acta Otorrinolaringol Esp.* 2015;66(5):264-268.
10. Montero Ruiz E, Rebollar Merino A, Castejón Cervero MA, Barbero Allende JM, Culebras López A, García Sánchez M. Effect of co-management with Internal Medicine on hospital stay in Ophthalmology. *Arch Soc Esp Oftalmol.* 2015;90(6):253-256.
11. Iberti CT, Briones A, Gabriel E, Dunn AS. Hospitalist-vascular surgery comanagement: effects on complications and mortality. *Hosp Pract (1995).* 2016;44(5):233-236.
12. Noticewala MS, Swart E, Shah RP, Macaulay W, Geller JA. First Place Award: Multidisciplinary care of the hip fracture patient: A case control analysis of differing treatment protocols. *Curr Orthop Pract.* 2016;27(4):346-350.
13. Rohatgi N, Loftus P, Grujic O, Cullen M, Hopkins J, Ahuja N. Surgical comanagement by hospitalists improves patient outcomes: a propensity score analysis. *Ann Surg.* 2016;264(2):275-282.
14. Soong C, Cram P, Chezar K, et al. Impact of an integrated hip fracture inpatient program on length of stay and costs. *J Orthop Trauma.* 2016;30(12):647-652.
15. Rohatgi N, Wei PH, Grujic O, Ahuja N. Surgical comanagement by hospitalists in colorectal surgery. *J Am Coll Surg.* 2018;227:404-410.

© 2020 Shaw M et al. *JAMA Network Open.*

eTable 2. Risk of Bias for Included Studies for the Outcomes of Length of Stay, Mortality, and Readmissions

Red indicates high risk, orange moderate and green low risk.

Study	Randomization process	Confounding	Selection	Classification of interventions	Deviation from intended interventions	Missing data	Measurement of outcomes	Selection of reported result	Overall risk of bias
Zuckerman (1991)									
Macpherson (1994)									
Huddleston (2004)									
Pinzur (2006)									
Salottolo (2009)									
Auerbach (2010)									
Della Rocca (2013)									
Montero Ruiz (2015)									
Montero Ruiz (2015)									
Iberti (2016)									
Noticewala (2016)									
Rohatgi (2016)									
Soong (2016)									
Rohatgi (2018)									

© 2020 Shaw M et al. *JAMA Network Open.*

eFigure 1. Forest Plot for Unadjusted Length of Stay by Physician-Only or Multidisciplinary Team (MDT) Model

	Intervention			Comparator				Mean Difference	
**Study or Subgroup**	**Mean**	**SD**	**Total**	**Mean**	**SD**	**Total**	**Weight**	**IV, Random, 95% CI**	**Year**
**1.1.1 physician only**									
Macpherson 1994	19.7	18.2	79	27.2	18.2	86	3.1%	-7.50 [-13.06, -1.94]	1994
Salottolo 2009	4.15	2.04	261	3.64	1.91	239	19.0%	0.51 [0.16, 0.86]	2009
Montero Ruiz 2015a ENT	3.5	9.05	642	2.8	9.62	987	16.9%	0.70 [-0.22, 1.62]	2014
**Subtotal (95% CI)**			**982**			**1312**	**39.0%**	**0.21 [-1.05, 1.48]**	
Heterogeneity: Tau² = 0.75; Chi² = 8.14, df = 2 (P = 0.02); I² = 75%									
Test for overall effect: Z = 0.33 (P = 0.74)									
**1.1.2 MDT**									
Della Rocca 2013	7.1	4.33	115	9.9	9.41	31	6.5%	-2.80 [-6.21, 0.61]	2013
Noticewala 2016	8.2	4.1	129	10.7	13.6	138	9.9%	-2.50 [-4.88, -0.12]	2016
Iberti 2016	6.1	6.17	1487	5.1	6.17	944	18.6%	1.00 [0.50, 1.50]	2016
Soong 2016	11.9	13.7	331	18.2	18.4	240	8.4%	-6.30 [-9.06, -3.54]	2016
Rohatgi 2018	6.3	8.3	1062	7.6	8.5	938	17.7%	-1.30 [-2.04, -0.56]	2018
**Subtotal (95% CI)**			**3124**			**2291**	**61.0%**	**-2.03 [-4.05, -0.01]**	
Heterogeneity: Tau² = 4.20; Chi² = 53.99, df = 4 (P < 0.00001); I² = 93%									
Test for overall effect: Z = 1.97 (P = 0.05)									
**Total (95% CI)**			**4106**			**3603**	**100.0%**	**-1.02 [-2.09, 0.04]**	
Heterogeneity: Tau² = 1.53; Chi² = 65.71, df = 7 (P < 0.00001); I² = 89%									
Test for overall effect: Z = 1.88 (P = 0.06)									
Test for subgroup differences: Chi² = 3.42, df = 1 (P = 0.06), I² = 70.7%									

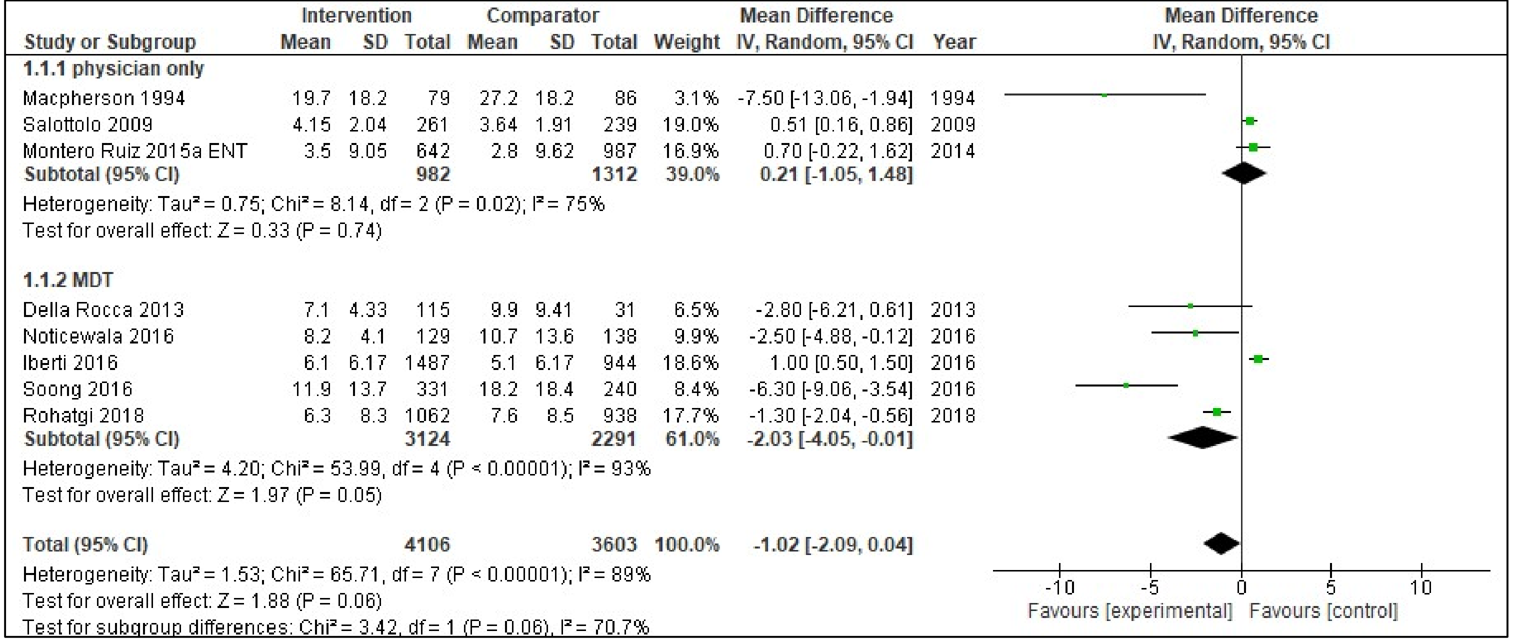

© 2020 Shaw M et al. *JAMA Network Open.*

eFigure 2. Forest Plot for Unadjusted Length of Stay by Elective or Emergency Status

Study or Subgroup	Intervention Mean	Intervention SD	Intervention Total	Comparator Mean	Comparator SD	Comparator Total	Weight	Mean Difference IV, Random, 95% CI	Year
**1.9.1 elective**									
Macpherson 1994	19.7	18.2	79	27.2	18.2	86	3.1%	-7.50 [-13.06, -1.94]	1994
Montero Ruiz 2015a ENT	3.5	9.05	642	2.8	9.62	987	16.9%	0.70 [-0.22, 1.62]	2014
Iberti 2016	6.1	6.17	1487	5.1	6.17	944	18.6%	1.00 [0.50, 1.50]	2016
Rohatgi 2018	6.3	8.3	1062	7.6	8.5	938	17.7%	-1.30 [-2.04, -0.56]	2018
**Subtotal (95% CI)**			**3270**			**2955**	**56.2%**	**-0.36 [-1.95, 1.22]**	
Heterogeneity: Tau² = 1.96; Chi² = 33.76, df = 3 (P < 0.00001); I² = 91%									
Test for overall effect: Z = 0.45 (P = 0.65)									
**1.9.2 emergency**									
Salottolo 2009	4.15	2.04	261	3.64	1.91	239	19.0%	0.51 [0.16, 0.86]	2009
Della Rocca 2013	7.1	4.33	115	9.9	9.41	31	6.5%	-2.80 [-6.21, 0.61]	2013
Soong 2016	11.9	13.7	331	18.2	18.4	240	8.4%	-6.30 [-9.06, -3.54]	2016
Noticewala 2016	8.2	4.1	129	10.7	13.6	138	9.9%	-2.50 [-4.88, -0.12]	2016
**Subtotal (95% CI)**			**836**			**648**	**43.8%**	**-2.61 [-5.92, 0.69]**	
Heterogeneity: Tau² = 9.84; Chi² = 31.95, df = 3 (P < 0.00001); I² = 91%									
Test for overall effect: Z = 1.55 (P = 0.12)									
**Total (95% CI)**			**4106**			**3603**	**100.0%**	**-1.02 [-2.09, 0.04]**	
Heterogeneity: Tau² = 1.53; Chi² = 65.71, df = 7 (P < 0.00001); I² = 89%									
Test for overall effect: Z = 1.88 (P = 0.06)									
Test for subgroup differences: Chi² = 1.45, df = 1 (P = 0.23), I² = 31.0%									

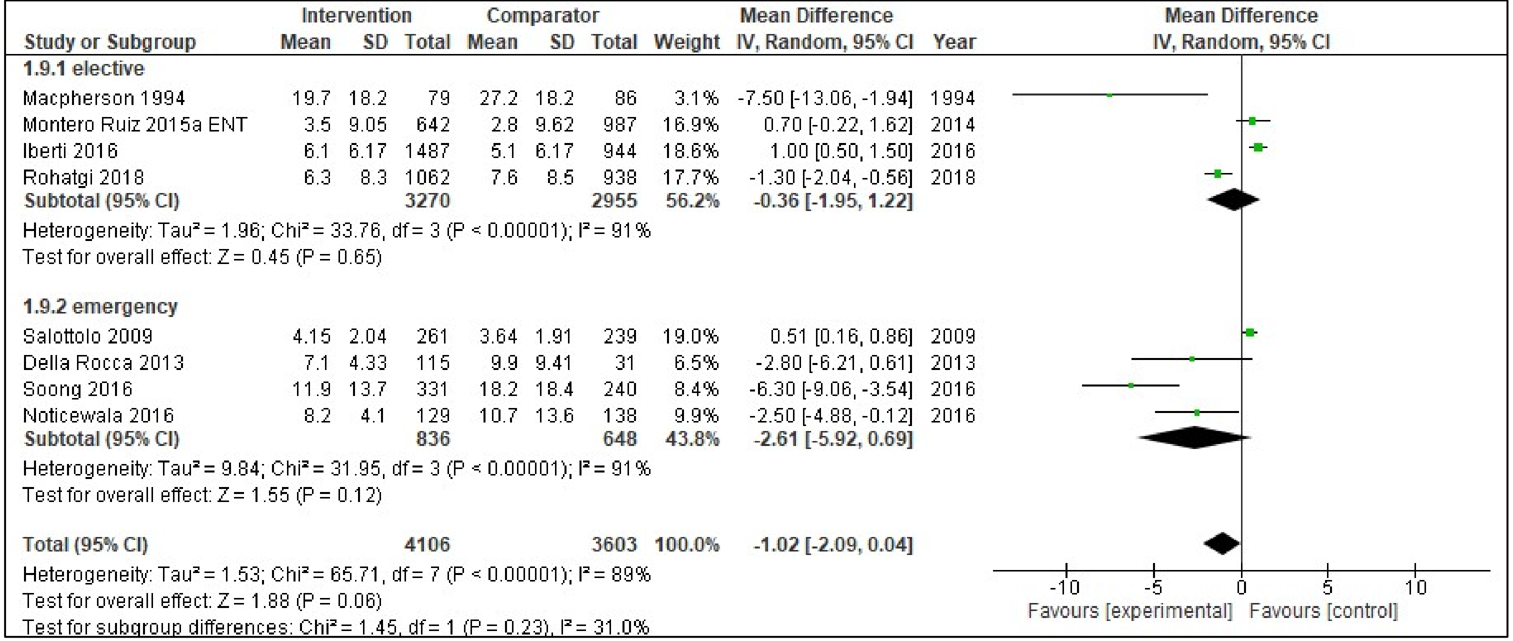

© 2020 Shaw M et al. *JAMA Network Open.*

eFigure 3. Funnel Plots for Length of Stay, Mortality, and Readmissions

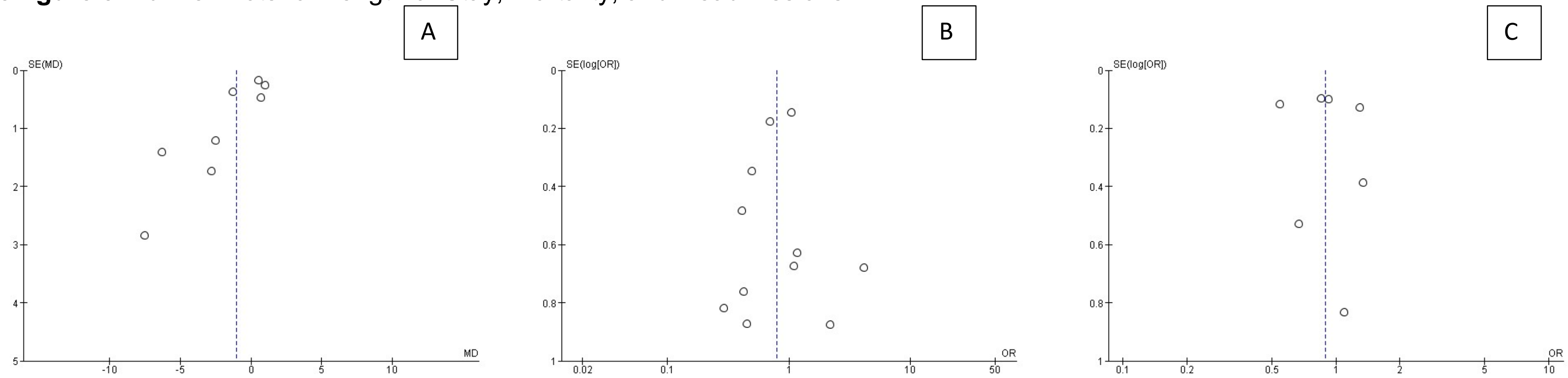

SE standard error, MD mean difference, OR odds ratio

© 2020 Shaw M et al. *JAMA Network Open.*

eFigure 4. Forest Plot for In-Hospital Mortality by Physician-Only or Multidisciplinary Team (MDT) Model

Study or Subgroup	Intervention Events	Intervention Total	Comparator Events	Comparator Total	Weight	Odds Ratio M-H, Random, 95% CI	Year
**1.8.1 physician only**							
Macpherson 1994	2	79	7	86	4.0%	0.29 [0.06, 1.46]	1994
Salottolo 2009	2	261	4	239	3.5%	0.45 [0.08, 2.50]	2009
Auerbach 2010	88	3393	104	4203	23.4%	1.05 [0.79, 1.40]	2010
Montero Ruiz 2015b Ophthal	0	244	0	345		Not estimable	2014
Montero Ruiz 2015a ENT	8	642	3	987	5.4%	4.14 [1.09, 15.66]	2014
**Subtotal (95% CI)**		**4619**		**5860**	**36.3%**	**0.98 [0.40, 2.41]**	
Total events	100		118				
Heterogeneity: Tau² = 0.48; Chi² = 7.45, df = 3 (P = 0.06); I² = 60%							
Test for overall effect: Z = 0.05 (P = 0.96)							
**1.8.2 MDT**							
Zuckerman 1992	25	431	3	60	6.1%	1.17 [0.34, 4.00]	1991
Della Rocca 2013	5	115	3	31	4.5%	0.42 [0.10, 1.88]	2013
Soong 2016	7	331	12	240	9.0%	0.41 [0.16, 1.06]	2016
Rohatgi 2016	40	4650	173	14156	21.8%	0.70 [0.50, 0.99]	2016
Noticewala 2016	4	129	2	138	3.5%	2.18 [0.39, 12.09]	2016
Iberti 2016	15	1487	19	944	13.3%	0.50 [0.25, 0.98]	2016
Rohatgi 2018	5	1062	4	938	5.5%	1.10 [0.30, 4.13]	2018
**Subtotal (95% CI)**		**8205**		**16507**	**63.7%**	**0.67 [0.51, 0.88]**	
Total events	101		216				
Heterogeneity: Tau² = 0.00; Chi² = 5.36, df = 6 (P = 0.50); I² = 0%							
Test for overall effect: Z = 2.88 (P = 0.004)							
**Total (95% CI)**		**12824**		**22367**	**100.0%**	**0.79 [0.56, 1.11]**	
Total events	201		334				
Heterogeneity: Tau² = 0.11; Chi² = 17.95, df = 10 (P = 0.06); I² = 44%							
Test for overall effect: Z = 1.34 (P = 0.18)							
Test for subgroup differences: Chi² = 0.62, df = 1 (P = 0.43), I² = 0%							

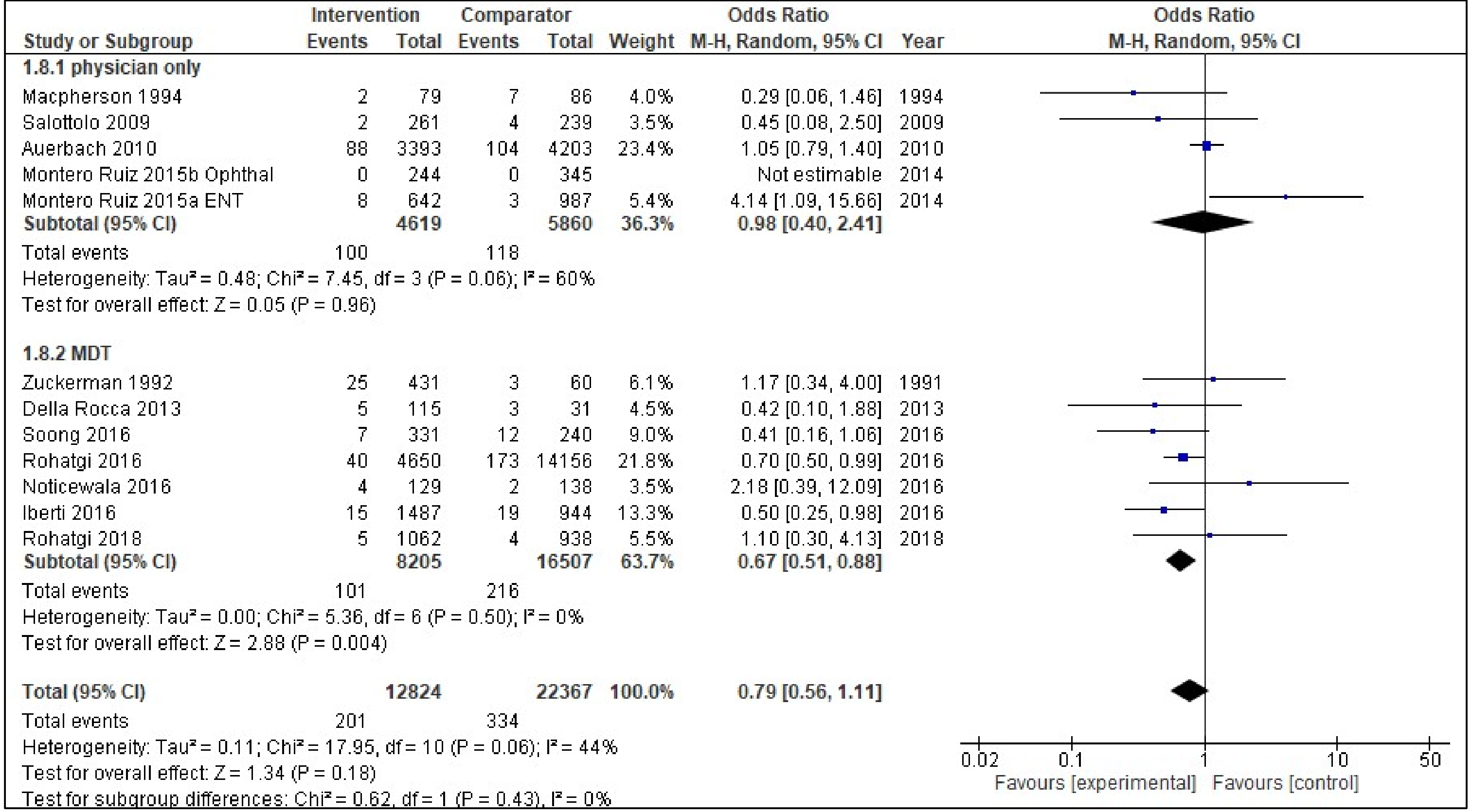

© 2020 Shaw M et al. *JAMA Network Open.*

eFigure 5. Forest Plot for In-Hospital Mortality by Elective or Emergency Status

Study or Subgroup	Intervention Events	Intervention Total	Comparator Events	Comparator Total	Weight	Odds Ratio M-H, Random, 95% CI	Year
**1.10.1 elective**							
Macpherson 1994	2	79	7	86	4.0%	0.29 [0.06, 1.46]	1994
Auerbach 2010	88	3393	104	4203	23.4%	1.05 [0.79, 1.40]	2010
Montero Ruiz 2015a ENT	8	642	3	987	5.4%	4.14 [1.09, 15.66]	2014
Montero Ruiz 2015b Ophthal	0	244	0	345		Not estimable	2014
Iberti 2016	15	1487	19	944	13.3%	0.50 [0.25, 0.98]	2016
Rohatgi 2016	40	4650	173	14156	21.8%	0.70 [0.50, 0.99]	2016
Rohatgi 2018	5	1062	4	938	5.5%	1.10 [0.30, 4.13]	2018
**Subtotal (95% CI)**		**11557**		**21659**	**73.4%**	**0.84 [0.55, 1.30]**	
Total events	158		310				
Heterogeneity: Tau² = 0.14; Chi² = 12.91, df = 5 (P = 0.02); I² = 61%							
Test for overall effect: Z = 0.77 (P = 0.44)							
**1.10.2 emergency**							
Zuckerman 1992	25	431	3	60	6.1%	1.17 [0.34, 4.00]	1991
Salottolo 2009	2	261	4	239	3.5%	0.45 [0.08, 2.50]	2009
Della Rocca 2013	5	115	3	31	4.5%	0.42 [0.10, 1.88]	2013
Noticewala 2016	4	129	2	138	3.5%	2.18 [0.39, 12.09]	2016
Soong 2016	7	331	12	240	9.0%	0.41 [0.16, 1.06]	2016
**Subtotal (95% CI)**		**1267**		**708**	**26.6%**	**0.65 [0.35, 1.19]**	
Total events	43		24				
Heterogeneity: Tau² = 0.02; Chi² = 4.18, df = 4 (P = 0.38); I² = 4%							
Test for overall effect: Z = 1.41 (P = 0.16)							
**Total (95% CI)**		**12824**		**22367**	**100.0%**	**0.79 [0.56, 1.11]**	
Total events	201		334				
Heterogeneity: Tau² = 0.11; Chi² = 17.95, df = 10 (P = 0.06); I² = 44%							
Test for overall effect: Z = 1.34 (P = 0.18)							
Test for subgroup differences: Chi² = 0.49, df = 1 (P = 0.49), I² = 0%							

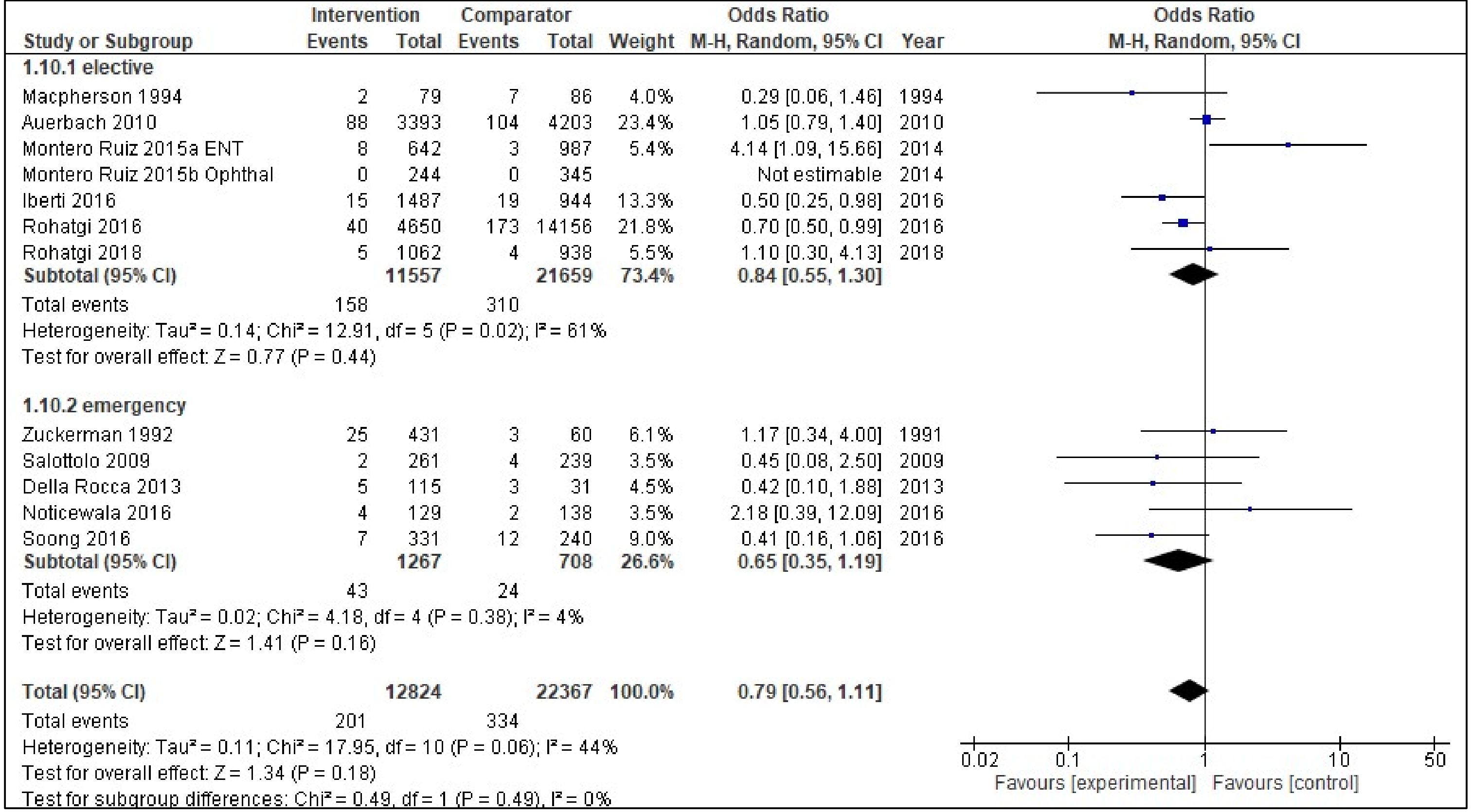

© 2020 Shaw M et al. *JAMA Network Open.*

eFigure 6. Forest Plot for 30-Day Readmissions

Study or Subgroup	Intervention Events	Intervention Total	Comparator Events	Comparator Total	Weight	Odds Ratio IV, Random, 95% CI	Year
Macpherson 1994	3	79	3	86	2.5%	1.09 [0.21, 5.58]	1994
Auerbach 2010	192	3393	277	4203	21.6%	0.85 [0.70, 1.03]	2010
Della Rocca 2013	16	115	6	31	5.4%	0.67 [0.24, 1.90]	2013
Soong 2016	20	331	11	240	8.4%	1.34 [0.63, 2.85]	2016
Iberti 2016	306	1487	207	944	21.4%	0.92 [0.76, 1.13]	2016
Rohatgi 2016	88	4650	481	14156	20.6%	0.55 [0.44, 0.69]	2016
Rohatgi 2018	172	1062	122	938	20.1%	1.29 [1.01, 1.66]	2018
**Total (95% CI)**		**11117**		**20598**	**100.0%**	**0.89 [0.68, 1.16]**	
Total events	797		1107				

Heterogeneity: Tau² = 0.08; Chi² = 26.99, df = 6 (P = 0.0001); I² = 78%

Test for overall effect: Z = 0.86 (P = 0.39)

Odds Ratio
IV, Random, 95% CI

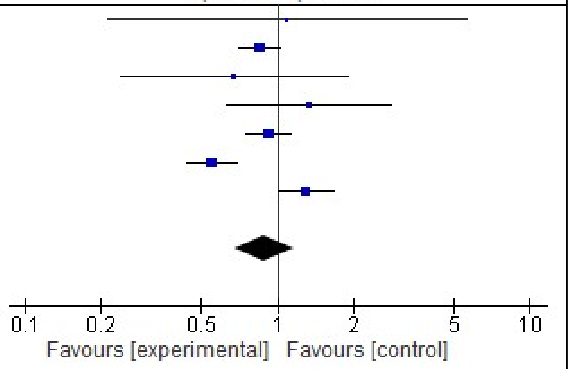

© 2020 Shaw M et al. *JAMA Network Open.*
